# Carbon Nanomaterials: A New Sustainable Solution to Reduce the Emerging Environmental Pollution of Turbomachinery Noise and Vibration

**DOI:** 10.3389/fchem.2020.00683

**Published:** 2020-08-20

**Authors:** Xiao Qi Jia, Song Yu Li, Hong Jiang Miu, Tuo Yang, Kun Rao, Dong Yang Wu, Bao Ling Cui, Jun Lang Ou, Zu Chao Zhu

**Affiliations:** ^1^Key Laboratory of Fluid Transmission Technology of Zhejiang Province, Zhejiang Sci-Tech University, Hangzhou, China; ^2^Hangzhou Dalu Industry Co., Ltd, Hangzhou, China; ^3^Hangzhou Oxygen Plant Group Co., Ltd, Hangzhou, China

**Keywords:** carbon nanomaterials, emerging environmental pollution, vibration and noise reduction, sustainability, turbomachinery

## Abstract

The vibration and noise that resulted from turbomachinery, such as fans, compressors, and centrifugal pumps, are known to bring considerable disturbance and pollution to the machine itself, the environment, and the operators. Hence, how to cope with the vibration and noise has become a recent research focus. With the advancement of materials science, more and more new nanomaterials have been applied in the field of noise and vibration reduction. To be specific, carbon-based nanomaterials, such as carbon fibers, carbon nanotubes, and graphenes, have achieved outstanding results. Carbon nanocomposites, such as carbon nanofibers, carbon nanotubes, and graphenes, are characterized by their low densities, high strengths, and high elastic moduli, all of which made carbon nanocomposites the most promising vibration and noise-reduction composites, thanks to their damping properties, compatibilities, noise and vibration absorption qualities, and wide wave-absorbing frequency bands. In light of this, this paper summarizes the progresses and application prospects of such carbon nanocomposites as carbon nanofibers, carbon nanotubes, and graphenes in the field of turbomachinery vibration and noise reduction.

## Introduction

Noise pollution is deemed as one of the four major types of environmental pollution apart from air, water, and solid waste pollutions, and it poses a significant influence on not only human health but also environmental damage. Noise can affect the nervous system, endanger human health, destroy the ecosystem, and pollute the environment, leading to the deaths or even extinctions of living things (Stephen and Mark, 2003; Francis et al., 2009; Mehdi et al., 2011). Turbomachinery, such as car engines, large fans, compressors, and centrifugal pumps, has become a major source of noise pollution (Forouharmajd et al., 2012; Gabbert et al., 2014; Sun et al., 2020a). Because of the need for turbomachinery to run continuously (Jia et al., 2019; Chen et al., 2020) the noise pollution caused by turbomachinery also continues to rise, dramatically increasing the sound pressure to a point that exceeds the pain threshold surmountable by humans and animals alike. Noise pollution can not only cause detriment to people's hearing, sleep quality, psychology, and environment but also accelerate mechanical breakdown and affect the precision as well as service life of the equipment. Because turbomachinery is being developed along the path of larger power, higher rotation speed, and higher pressure, the noise pollution problems also continue to exacerbate. As such, these noise pollution problems have become one of the many environmental pollution problems that need to be solved urgently. Traditionally, turbomachinery's noise and vibration reductions have been handled from the perspectives of vibration source and transmission path using passive control measures (Davies et al., 1982; Elsukov et al., 1984; Bies et al., 1996; Choi et al., 2003; Coleman and Remington, 2007). With the advancement of materials science nowadays, more and more new materials have been utilized (Sarkar et al., 2009; Shukla et al., 2015; Isaias et al., 2017; Liu et al., 2019; Sun et al., 2020b). Carbon nanocomposites, such as carbon nanofibers, carbon nanotubes, and graphenes, are characterized by low density, high strength, and high elastic modulus, which give carbon nanocomposites their damping properties, compatibilities, noise and vibration absorption properties, and wide wave-absorbing frequency bands (Baughman, 2002; Young et al., 2012; Nieto et al., 2016), making them the most promising vibration and noise reduction composites. Existing practical research on carbon nanocomposites in turbomachinery vibration and noise reduction is predominantly predicated upon the damping properties as well as sound and vibration absorptions. In this paper, progresses and application prospects of carbon nanocomposites in the field of turbomachinery vibration and noise reduction were discussed in two ways—damping property and sound absorption property of carbon nanocomposites.

## Vibration and Noise Reduction Based on the Damping Property

The approach of reducing vibration and noise based on the damping property is one of the most critical means by which vibration and noise can be reduced in machinery (Hang et al., 1993; Lavernia et al., 1995). In this approach, the vibration energy of the mechanical system is converted into other forms of energy (such as thermal energy, and deformation energy) so that the system can be recovered to the pre-stimulated state as quickly as possible. Currently, popular damping materials include viscoelastic damping materials, damping composite materials, metal damping materials, and intelligent damping materials. Carbon nanocomposites, such as carbon nanofibers, carbon nanotubes, and graphenes, have become the most promising composites for vibration and noise reduction due to their low densities, high strengths, and high elastic moduli.

Carbon fibers have been more and more widely used in the fields of aerospace and petrochemical and medical treatment (Sheehan et al., 1994; Park and Kim, 2010), owing to their unique stabilities, elastic moduli, strengths, stiffness, thermal conductivities, and lightweightness (Ruland, 1990; Keiji et al., 2004; Wu et al., 2010). Nevertheless, the high stiffness of carbon-fiber composite materials could lead to relatively low damping performance, in which case original cracks will be quickly propagated under the actions of periodic loads and external shocks, eventually resulting in vibration, fatigue, and failure (Khan et al., 2011; Fan et al., 2016). Hence, effective measures should be taken to enhance the damping properties of carbon fiber materials. Based on the laminate board structure of carbon fiber acoustic metamaterial, He et al. (2017) not only effectively improved the damping property of carbon fiber materials but also achieved an effective vibration suppression effect by applying the laminate board of acoustic metamaterials to an automobile door design. Li et al. (2017) found that laying flax fibers on the outermost layer of the composite can effectively improve the damping property of the carbon fiber-reinforced composite after studying the influence of flax fiber stacking sequence and carbon nanotube additives on the damping and mechanical properties of carbon fiber-reinforced composites. Lin et al. (1984) provided an important guidance for the design of carbon fiber composite materials for vibration and noise reduction after investigating the intrinsic frequency and specific damping capacity of carbon fiber composite plates using various vibration models. According to Han's research (Han and Chung, 2012), mixing discontinuous particulate-shaped and fibrous fillers between carbon fiber sheets can effectively enhance the damping performance of carbon fiber materials.

Carbon nanotube was first discovered by Lijima (1991) in 1991. Every carbon atom in a carbon nanotube is connected to three adjacent carbon atoms, forming a hexagonal grid structure (Henning and Salama, 1998). In 1994, the carbon nanotube was used by Ajayan et al. (1994) as reinforcement material for polymer nanocomposites for the first time. According to Zhang's research (Zhang et al., 2011), the mechanical properties of the carbon nanotube is much better than that of carbon fiber. Carbon nanotubes can increase the damping properties of materials to a certain extent and hence facilitate vibration and noise reduction of mechanical systems (Cao et al., 2005; Liu et al., 2008; Zhang et al., 2009). Kundalwal and Meguid (2015) studied the effects of carbon nanotubes on the composite's active constrained-layer damping and discovered that the damping performance of multifunctional nanocomposite structures can be effectively improved with wavy carbon nanotubes. Ma et al. (1998) prepared carbon nanotube/nano-silicon carbide ceramic matrix composites via the hot pressing approach and found that their flexural strengths and fracture toughness were increased by 10% in comparison to nano-silicon carbide ceramics prepared under the same conditions. Fereidoon and Ashoory (2010) studied the damping properties of various carbon nanotube polymer composites and pointed out that mixing carbon nanotube fillers in the resin can improve the damping property. Johnson et al. (2011) reported that the damping ratio of fiber composites can be well increased after adding carbon nanotubes to fiber composites. Zhou et al. (2004) found that carbon nanotube fillers can increase the stick–slip friction on the surface of nanotubes, thus increasing the material damping. A study by Suhr and Koratkar (2008) indicated that the friction energy dissipation generated by the sliding of carbon nanotubes on the polycarbonate matrix can increase the damping ratio of materials. Furthermore, results obtained from the study of Cao et al. (2005) showed that the overlapped position of the carbon nanotube–carbon nanotube enhances the viscous effect in the matrix, presenting a significant damping enhancement effect.

Mittal and Chaudhry (2015) concluded that graphenes can raise the damping properties of nanocomposites by approximately 70% based on their study concerning the relationship between the mechanical damping behaviors of graphene-reinforced polymer nanocomposites and the surface morphologies of graphene nanosheets. Moreover, rigorous analyses have also suggested that the graphene filler interface can reinforce the damping property of nanocomposites (Stankovich et al., 2006; Singh et al., 2011; Young et al., 2012). According to Kim's research (Kim et al., 2017), adding graphenes to polyurethane foams can contribute to the reduction of the polyfoam's cell size and the increase in the polyfoam's bending path, thereby enhancing its acoustic damping performance. Sudeshna et al. (2017) found that the water content of graphene oxides plays a critical role in the damping performance of graphene oxide films; specifically, the lower the water content, the higher the viscoelasticity, and the greater the damping ratio. Tang et al. (2014) studied the mechanical and damping properties of graphene oxide/epoxy resin composites and found that the impact strengths and the damping properties of the composites are significantly improved with the increase in graphene oxide content.

## Vibration and Noise Reduction Based on the Property of Sound and Vibration Absorption

Apart from utilizing materials with damping properties, those with noise and vibration absorption properties are also one of the main research focuses in the study of noise and vibration reduction using composite materials. The sound and vibration absorption properties of composites are such that different velocity gradients are generated from the energy of energy waves when sound and vibration waves propagate in a variety of media (such as air, and sound-absorbing materials). Meanwhile, movement between adjacent mediator molecules can produce viscous and friction forces, causing the energy wave to be dissipated after being converted into thermal energy (Kishor and Pawar, 2019). Along with the advancement of materials science, more and more composite materials have also been applied in the field of noise and vibration absorption (Dunne et al., 2017; Yang and Sheng, 2017; Zhao et al., 2018; Yang et al., 2019; Xie et al., 2020). Carbon nanocomposites, such as carbon nanofibers, carbon nanotubes, and graphenes, have become the most promising vibration and noise absorption composites, owing to their favorable compatibilities, excellent sound and vibration absorption properties, and wide wave absorption frequency band.

Carbon nanofiber is a kind of high-performance and sound-absorbing multiporous fiber material (Rodrfguez-Mirasol et al., 1993) that consists of a large number of interpenetrable micropores. Chen and Jiang (Chen and Jiang, 2007) found that the sound absorption performance of the activated carbon fiber composite is superior to that of other composites. Based on this, Shen and Jiang (2016), Shen and Jiang (2014a,b) established a sound absorption theory model of activated carbon fiber materials by referring to the circular tube theory model and tested the sound absorption coefficient of the activated carbon fiber sensed in the frequency range of 250–6,300 Hz using a dual-channel impedance tube acoustic analyzer. The result showed that the established sound absorption theory model of activated carbon fiber materials can provide theoretical and technical supports for designing and developing activated-carbon-fiber-based sound absorption materials.

Carbon nanotubes with a high length–width ratio can present good sound absorption performance at low concentrations (Khosla and Gray, 2009). Ayub et al. (2018) found that sound absorptions are associated with the molecular interactions between sound waves and nanomaterials, which take place under a very high frequency. What is more, they (Ayub et al., 2017) also found that a 3-millimeter-thick carbon nanolayer can provide up to 10% of sound absorption in the frequency range of 0.125–4 kHz. According to the study carried out by Bandarian et al. (2011), the porous wall carbon nanotube can reinforce the sound absorption capability of materials. It has also been found from some studies (Verdejo et al., 2009; Willemsen and Rao, 2015) that adding a low concentration of carbon nanotubes can improve the sound absorption performance of polyurethane foam. Based on the study performed by Gu et al. (2018), the addition of porous wall carbon nanotubes can effectively improve the underwater sound absorption performance of polyurethane coatings. On this basis, Kabir et al. (2020) also added surfactants and carboxyl-functionalized multiwalled carbon nanotubes to the polydimethylsiloxane nanocomposite membrane for underwater sound absorption to improve the sound absorption performance of the nanocomposites.

Since the interface interaction between graphenes and the matrix material could have a significant impact on the morphology, mechanical property, and electrical property of the composite, the addition of graphene will exert a certain modifying effect on metals, semiconductors, ceramics, polymers, and biomaterials. Because of such, the material constituted by the single-layer carbon atoms can be extensively used in polymer composites (Kim et al., 2010), thin film displays (Bae et al., 2010), and semiconductor devices (Liu et al., 2017). Moreover, because of graphene's high strength and modulus, the modifier, which is supposed to turn graphene into polymer materials, is also responsible for giving the prepared composite high rigidity and surface density, hence contributing positively to the acoustic property of the composite. Zhang and Xu (2011) predicted the strong sound absorption properties of surface acoustic waves in a wide frequency range below terahertz on the basis of studying the sound absorption of graphene under the effect of sound waves. Qamoshi and Rasuli (2016) enhanced the sound absorption capacity of polymers at a specific frequency upon the addition of graphene oxides to the carbon fiber polymers. Nine et al. (2017) enhanced the noise absorption capacity of a honeycomb grid structure by controlling the porosity and sinuosity of oxidized graphene sheets. Carolina et al. (2019) found through their study that reduced graphene oxide can improve the thermal stability and sound absorption coefficient of aerogels, creating a new research orientation for the preparation of emerging materials with excellent thermal insulation and sound absorption properties.

## Conclusion and Perspective

Since turbomachinery is the most widely used general-purpose machine in mechanical engineering, its vibratory noise problem has always been seen as a major issue and, hence, a research focus. In order to address this problem, researchers have studied vibration and noise reduction based on the damping performance and sound absorption properties of such carbon nanocomposites as carbon nanofibers, carbon nanotubes, and graphenes. Due to the late start of its development and application, carbon nanocomposites still have deficiencies in comparison to traditional metal materials in terms of achieving high-quality continuous production. Furthermore, many materials are still under development, while the growth mechanism of some carbon nanomaterials is still unclear, and the structure of carbon composites cannot be adjusted and controlled arbitrarily. Hence, it is imperative to further study carbon composites with vibration and noise reduction properties as well as wearproof corrosion characteristics. In this way, carbon nanocomposites with special functions can be developed by giving full play to their excellent properties. Meanwhile, apart from damping as well as sound and vibration properties, multifunctional carbon nanocomposites featuring high temperature resistance, corrosion resistance, and wear resistance should be also developed, due to their prospects in becoming a dominant research focus and development trend pertaining to turbomachinery. In the future, the application of carbon nanocomposites in surface coatings, low sound and vibration composites, compound material bearing in the field of turbomachinery vibration, and noise reduction will become the hotspot.

## Author Contributions

XJ and ZZ contributed to the conception of the study. XJ and SL wrote the article. HM, TY, DW, and JO searched the information. SL and BC edited the article. All authors contributed to the article and approved the submitted version.

## Conflict of Interest

HM, TY, and KR were employed by the company Hangzhou Dalu Industry Co., Ltd. DW was employed by the company Hangzhou Oxygen Plant Group Co., Ltd. The remaining authors declare that the research was conducted in the absence of any commercial or financial relationships that could be construed as a potential conflict of interest.
